# Effectiveness of four interventions in improving community health workers’ performance in western Kenya: a quasi-experimental difference-in-differences study using a longitudinal data

**DOI:** 10.1017/S1463423622000135

**Published:** 2022-03-25

**Authors:** Yoshito Kawakatsu, Tomohiko Sugishita, Hirotsugu Aiga, Kennedy Oruenjo, Steve Wakhule, Sumihisa Honda

**Affiliations:** 1 Department of Global Health, University of Washington, USA; 2 Graduate School of Biomedical Sciences, Nagasaki University, Nagasaki, Japan; 3 School of Medicine, Tokyo Women’s Medical University, Japan; 4 Department of Global Health, School of Medicine and Health Sciences, The George Washington University, Washington, DC, USA; 5 School of Tropical Medicine and Global Health, Nagasaki University, Nagasaki, Japan; 6 Ministry of Health, Siaya, Kenya

**Keywords:** community health worker, effectiveness, financial incentive, nonfinancial incentive, performance, supervision, training

## Abstract

**Background:**

Community health workers (CHWs) are up-front health workers delivering the most effective life-saving health services to communities. They are the key driver to achieve Universal Health Coverage. However, maintaining CHWs’ performance is one of the challenges in sustaining their effectiveness. This article assessed the effectiveness of the four interventions and their combinations on the CHWs’ performance in terms of health knowledge, job satisfaction, and household coverage.

**Methods:**

We used the longitudinal survey data collected in western Kenya. Our study participants were the representative of all CHWs working in the four districts, Kenya. The four types of interventions were composed of a basic core intervention (i.e., refresher training with/without defaulter tracing) and three supplementary interventions (i.e., provision of a bicycle, frequent supportive supervision, and financial incentives). We performed the three fixed-effect models to assess the effectiveness of the four interventions and their combinations on the three performance indicators.

**Results:**

Three single and combination interventions significantly increased CHWs’ health knowledge: refresher training only [Coef.: 48.43, 95% CI: 42.09–54.76, *P* < 0.001]; refresher training plus defaulter-tracing [Coef.: 38.80, 95% CI: 32.71–44.90, *P* < 0.001]; combination of refresher training plus defaulter-tracing and frequent supervision [Coef.: 17.02, 95% CI: 7.90–26.15, *P* < 0.001]. Financial support was the only intervention that significantly increased job satisfaction among CHWs [Coef.: 4.97, 95% CI: 0.20–9.75, *P* = 0.041]. There was no single intervention that significantly increased household coverage. Yet, the combinations of the interventions significantly increased household coverage.

**Conclusions:**

There was no single intervention to improve all the aspects of CHWs’ performance. The refresher training significantly improved their health knowledge, while financial incentive enhanced the level of their job satisfaction. The combinations of regular refresher training and other intervention(s) are the recommended as the effective interventions in improving and further sustaining CHWs’ performance.

## Background

Community health worker (CHW) program is one of the effective interventions in improving maternal and child health (MCH) outcomes (Haines *et al.*, 2007; Global Health Workforce Alliance and WHO, 2010; Lassi *et al*., 2010; Lewin *et al*., 2010; Perry *et al*., 2014). A great number of low- and middle-income countries have been implementing the CHW programs at a large scale, to fill the MCH-related health workforce deficiencies at community level. Yet, a majority of the CHW programs encounter two types of major operational challenges that largely stem from the limitations in keeping CHWs adequately motivated. First, attrition of CHWs attributable to their poor motivation is often observed. CHW attrition rate significantly varies between countries, for example, from 5% in Nepal to 74% in Bangladesh (Nkonki *et al*., 2011). High attrition rates result in frequent replacements of CHWs overtime that lead not only to an increase in operational costs of the program (i.e., recruitment, training, deployment, and supportive supervision for freshmen CHWs) but also to a loss of opportunities for CHWs to get more professionally experienced and build greater trust relationship between community people and themselves (Bhattacharyya *et al*., 2001). Second, CHWs’ performances do not necessarily improve as expected in terms of their productivity (Jaskiewicz and Tulenko, 2012) and effectiveness (Kok *et al*., 2014). While some earlier studies reported intensive training as an effective intervention for enhancing CHWs’ motivation and thereby performances (Harvey *et al.*, 2008; Kane *et al.*, 2010; Lopes *et al*., 2014), others reported supportive supervision as a recommended intervention for their poor performances (Kane *et al*., 2010; Laínez *et al*., 2012). Financial incentives, such as performance-based financing and remunerations in the form not of ad-hoc honorarium but of monthly salary, have been recently drawing a greater attention as an effective intervention that would help reduce CHWs’ attrition and improve CHWs’ performances (Kironde and Klaasen, 2002; Rahman *et al*., 2010; Miller *et al*., 2014; Zheng *et al.*, 2019), by increasing their motivation. Yet, pros and cons on the way of increasing CHWs’ motivation through financial incentives were reported in earlier studies (Kironde and Bajunirwe, 2002; Glenton *et al.*, 2010; Ormel *et al*., 2019). For instance, Glenton *et al*. (2010) reported that financial incentives would, in the long run, damage CHWs’ intrinsic motivations that often serve as the major reason for their participations in community-based activities (Mkandawire and Muula, 2005; Amare, 2009).

In Kenya, the Community Health Strategy was developed and launched to strengthen community health activities in 2006. In the strategy, a community unit (CU) composed of CHWs, community health extension workers (CHEWs), and community health committee (CHC) is defined as Level-1 health service delivery unit (Ministry of Health Kenya, 2005; Ministry of Public Health and Sanitation, 2008). In the strategy revised in 2010, monthly remuneration for CHWs was newly introduced and the standard number of households covered per CHW increased from 20 to 100. Yet, it is reality that these revised points, monthly remunerations in particular, were inadequately implemented because of poor planning and insufficient financing. Development partners and nongovernmental organisations (NGOs) have been providing the Kenyan Ministry of Health (MoH) with budgetary supports to financial and nonfinancial incentives for CHWs (e.g., remunerations, training, and supportive supervision). One of those external supports to developing the nonfinancial incentive system for CHWs was the Project for Strengthening Management for Health in Nyanza Province (the Project), funded by Japan International Cooperation Agency (JICA) and implemented jointly by JICA and the Kenyan MoH. The project implemented monthly refresher training for CHWs and defaulter tracing by CHWs, as the key nonfinancial incentive components for CHWs.

The study is aimed at estimating the effectiveness of four types of interventions for improving CHWs’ performances, using the longitudinal data collected for a cluster-randomized control trial in Nyanza Province, Kenya. The four types of interventions were composed of a basic core intervention (i.e., refresher training with/without defaulter tracing) and three supplementary interventions (i.e., provision of a bicycle, frequent supportive supervision, and financial incentives). We further estimated the effectiveness of combinations of basic core intervention and respective supplementary interventions.

## Methods

### Study sites

The study was conducted, all the 64 CUs established by 2011 in Gem, Kisumu West, Siaya, and Ugenya districts, Nyanza province, Kenya. A CU is set up per 5,000 population. Nyanza is one of the provinces recognized as both malaria (prevalence: 20–40%) and HIV (prevalence: 15.3%) endemic areas. Its major ethnic group is Luo, and the most frequently spoken language is Luo, followed by Swahili and English.

In Nyanza province, CHWs undertake regular household visits for the purpose of (i) providing health education sessions at each household; (ii) identifying women having danger signs during pregnancy and other households members’ health problems; (iii) referring them to the health facility linked to the CU; (iv) monitoring patients’ recovery process at households; and (v) collecting updated households’ sociodemographic and health data. They were not authorized to provide household members with any treatments.

### Datasets of the baseline and follow-up surveys

In this study, we analyzed the data previously collected in the baseline and follow-up surveys for the cluster randomized control trial (ISRCTN 18201040).

The baseline survey was conducted during the period from September to October 2011 in all the 64 CUs of Gem, Kisumu West districts, Siaya, and Ugenya districts, Nyanza Province, Kenya. First, the list of practicing CHWs was developed, at the time of the baseline survey. One of the criteria to become a CHW was the ability of speaking, reading, and writing English. A practicing CHW was defined as a CHW having been involved in any community health activities during the last three months. Second, after obtaining informed consents from all the 1,291 target CHWs practicing in the 64 CUs, a self-administered semi-structured questionnaire was completed in written-format by each one, to collect data on their sociodemographic and socioeconomic characteristics, health knowledge, and job satisfaction. The questions about financial incentives, nonfinancial incentives (i.e., provision of a bicycle), and frequencies of supervision undertaken by CHEWs were also asked in the questionnaire. Third, the list of mothers having children 12–23 months of age in the survey sites was developed. Fourth, to assess CHWs’ performances from clients’ perspective, 40 mothers were randomly selected per CU from the list. As a result, a total of 2,560 mothers (= 40 mothers x 64 CUs) were selected. Finally, locally recruited enumerators conducted interviews with those selected mothers, by using the structured questionnaire to assess the quantities of CHWs’ household visits by CHWs in each community.

The follow-up survey was conducted during the period from September to October of 2012. Upon the revision of the Community Health Strategies by the Kenyan MoH in 2010, the CHW program has been gradually implemented nationwide. In early 2012, Nyanza provincial department of health adjusted the number of households per CHW in response to revision of the strategy. As a result, the total number of CHWs was reduced from 1,291 at the baseline survey in 2011 to 691 at the follow-up survey in 2012. Of 691 self-administered questionnaires, 228 included missing data. Thus, the final number of CHWs having produced complete data at both baseline and follow-up was 463 (=691-228). In a similar manner to the baseline survey, a total of 2,560 randomly selected mothers (= 40 mothers x 64 CUs) were interviewed, by using the structured questionnaire in the follow-up survey.

### Interventions

The effectiveness of the four interventions was assessed in this study. Those four interventions were composed of a basic core intervention (i.e., refresher training with/without defaulter tracing) and three supplementary interventions (i.e., provision of bicycles to CHWs, >1 supportive supervision of CHWs per month, and financial incentives for CHWs). Of the four types of interventions, refresher training with/without defaulter tracing were implemented by the Project. All the 64 CUs were randomly assigned to three groups by type of basic core intervention for CHWs: (i) refresher training only (20 CUs); (ii) refresher training plus defaulter tracing (20 CUs); and (iii) control group (neither refresher training nor defaulter tracing (24 CUs). In the baseline and follow-up surveys, the enumerators were blinded to the random assignment process and results. The other three interventions were implemented as the activities by the organizations other than the project.

All the 302 CHWs in 40 CUs of refresher training group and refresher training plus group received either 1-day or 2-day refresher training on a monthly basis, consecutively during seven months from January to July of 2012. The mean total number of refresher training days per CHW was 11 days, that is, 1.6 days per month (=11/7). While CHWs were in principle volunteer workers being paid neither on a regular nor an ad-hoc basis, the MoH recommended that opportunities to have monthly regular refresher training be provided to CHWs in order for sustaining their motivation. Refresher training was implemented in a cascading manner. First, CHEWs were trained as supervisors of CHWs, by the district health officials responsible for community health. Second, CHEWs further trained CHWs on key topics related to basic community health. CHWs and the local leaders in their catchment communities are responsible for identifying and booking the training venues within their communities. This arrangement significantly helped save the training costs. The key topics related to basic community health addressed in refresher training include: (i) facilitation skills (i.e., overall facilitation and communication skills, leadership management and governance, couching and mentoring, time management, effective meeting, and proposal writing skills); (ii) case management (i.e., high impact health interventions, risk factors and danger signs during pregnancy, danger signs during childhood, case management of fever and diarrhea, and nutritional education); and (iii) data management (i.e., definitions of indicators in CHW monthly report, data cleaning methods, data analysis and interpretation methods, and data presentation skills). Each refresher training session addressed one or two of the aforementioned key topics. Since CHWs are responsible for health education for local populations of their catchment communities, facilitation skills are the essential expertise required for CHWs, particularly when implementing health education activities.

Defaulter tracing is the reminder system for tracking patients having missed their appointments for a series of maternal and child health services (e.g., antenatal care, child vaccinations, and child growth monitoring). When a patient has missed an upcoming health service appointment at the government health facilities, CHEWs add his/her name along with name of the community in which he/she resides as the landmark for CHWs, to the defaulter trace list. By referring to the list, CHWs trace those patients by physically visiting their households, remind them of missed appointment, and encourage them visit predetermined health facilities.

The MoH and its development partners provided CHWs with bicycles as the transport means for household visits and also as a nonfinancial incentive. CHEWs took supervisory responsibility for CHWs’ performances. The frequency of supportive supervisions varied by CHEWs’ own personal views and by availability of supports from the MoH and development partners. However, monthly supervision for CHWs is a standard frequency. More frequent supervision than monthly basis is a possible intervention to further improve CHWs’ performances. Financial incentives such as monthly remunerations and performance-based payments were provided to 81 CHWs by development partners under the agreement with the MoH, at the time of the follow-up survey in 2012. Note that the MoH did not provide CHWs in the study sites with monthly remunerations at the time of the baseline survey in 2011. Therefore, of 463 CHWs participating in both surveys, 81 were only those having received financial incentives. We collected the data on the other three interventions (i.e., provision of a bicycle, frequent supervision, and financial incentives) from CHWs during both surveys.

### Data analysis

This study employed the proxy indicators for CHWs’ performance as the outcome variables: (i) health knowledge; (ii) job satisfaction; and (iii) household coverage. The level of CHWs’ health knowledge was measured on immunization schedules, danger signs during pregnancy and childhood, risk factors for danger pregnancy, in the baseline survey. In the follow-up survey, the questions of body sites each vaccination injected, prevention methods for malaria, diarrhoea, and pneumonia, and high impact interventions were added to the topics of health knowledge to be measured, to estimate more comprehensive health knowledge among CHWs. CHWs’ health knowledge was scored jointly by plural clinical officers having completed medical education for at least three years. Spector’s questionnaire, a commonly cited questionnaire (Spector, 1985), was employed to measure job satisfaction, except its Pay and Promotion section, which was not applicable to CHWs. Household coverage was defined as the proportion of households visited by a CHW more than once per month to the total number of eligible households. To calculate household coverage of each CHW, the data collected from mothers during the baseline and follow-up surveys were used. All the three outcome variables were converted into proportions, that is, 0 to 100%.

Seven variables on CHWs’ sociodemographic and socioeconomic characteristics were employed as the confounding variables. Assuming their sociodemographic and socioeconomic characteristics either remained the same or would not change significantly during one year between two surveys, those data were collected only at the time of the baseline survey. Three of them were dichotomous variables, that is, sex (“male” or “female”), marital status (“married” or “others”), and the number of years of professional experiences (“less than 4 years” or “4 years or longer”). Age was categorized into three groups: “younger than 30 years of age”, “30–39 years of age”, and “40 years of age or older”. Similarly, education attainment was categorized into three levels: (i) no education or not completed primary education; (ii) primary education; or (iii) secondary education or higher. To categorize all CHWs into five groups from the poorest to the richest, their household wealth index was generated by the first principal component of principal component analysis using household structures, household assets, possession of animals, and monthly income. The level of availability of three types of sanitation and hygiene facilities at CHWs’ households (i.e., toilet, handwashing facility, and dish rack) were categorized into three groups: “no facility available”, “one or two facilities available”, “all three facilities available”.

All the statistical analyses were performed by using STATA (version 14, STATA Corporation, TX, USA). The level of statistical significance was set at 5%. Unadjusted effects of the four interventions were calculated, by comparing the difference between pre- and post-intervention of study group with that of control group, that is, unadjusted difference in difference (DID) estimator. To estimate the effectiveness of the four interventions and the combinations between the basic core intervention and three supplementary interventions, fixed-effect regressions were employed for respective outcome variables. For the fixed-effect regression models, the three outcome variables (i.e., health knowledge, job satisfaction, and household coverage) were used as the dependent variables, while seven CHWs’ background variables (i.e., sex, marital status, the number of years of professional experiences, age, education attainment, wealth quintiles, and availability of sanitation and hygiene facilities) were used as the independent variables. A fixed-effect regression controls both observed (i.e., the independent variables) and unobserved time-nonvarying variables (e.g., personal intrinsic characteristics and experiences), by subtracting the outcome at the follow-up survey from that at the baseline survey. Having assumed that the observed time-varying variables are independent of unobserved-time-varying variables, the coefficients estimated in the fixed effect regression models imply the magnitude of causality. The robust standard error was used for calculating a 95% confidence interval. We also performed univariate analyses to assess the association between the outcome and seven independent variables, which results are shown in the supplement Table 1. The association matrix between the intervention and control variables is also presented in the supplement Table 2.

**Table 1.** Sociodemographic and socioeconomic characteristics of community health workers (CHWs) at the baseline survey

Variables	Total (*n* = 463)	
	Number	%
Gender		
Female	364	78.6
Male	99	22.4
Age		
Younger than 30 years of age	49	10.6
30–39 years of age	191	41.3
40 years of age or older	223	48.2
Marital status		
Others	94	20.3
Married	369	79.7
Educational status		
No education or not completed primary education	43	9.3
Primary education	312	67.4
Secondary education or higher	108	23.3
Wealth index		
Poorest	94	20.3
Poor	55	11.9
Middle	97	21.0
Rich	123	26.6
Richest	94	20.3
Availability of sanitation facilities		
No facility available	49	10.6
One or two facilities available	143	30.9
All three facilities available	271	58.5
Working year as CHWs		
Less than 4 years	212	45.8
Four years or longer	251	54.2

**Table 2.** Unadjusted difference in difference estimates of the community health workers (CHWs’) performance indicators

Intervention groups versus control groups	Difference in difference		
	Health knowledge (%)	Job satisfaction (%)	Household coverage (%)
T1: Refresher training plus defaulter tracing versus C1: No training and defaulter tacing	45.66^***^	0.65	16.15^***^
T2: Refresher training versus C1: No training and defaulter tacing	50.09^***^	0.50	11.25^***^
B1: Owned a bicycle only at the follow-up versus C2: Not own	-1.99	-0.71	-1.01
B2: Owned a bicycle only at the baseline versus C2: Not own	-3.50	0.60	-6.05
B3: Possessed a bicycle at both surveys versus C2: Not owned	3.06	3.15	1.76
S1: More frequently supervised only at the follow-up versus C3: Supervised monthly or less	9.70^**^	0.45	13.04^***^
S2: More frequently supervised only at the baseline versus C3: Supervised monthly or less	-1.40	1.08	-6.40
S3: More frequently supervised at both surveys versus C3: Supervised monthly or less	17.83^*^	3.86	6.68
F1: Financial incentives at the follow-up versus C4: No financial incentives	6.15	0.75	15.10^***^

* *P* < 0.05.

** *P* < 0.01.

*** *P* < 0.001.

## Results

Table 1 shows sociodemographic and socioeconomic characteristics of 463 CHWs having fully responded to both the baseline and follow-up survey questionnaires. A majority of them were female (78.6%) and married (79.7%). Two hundred fifty-one (54.2%) of them had been working as CHWs for four years or longer.

**Figure 1.**
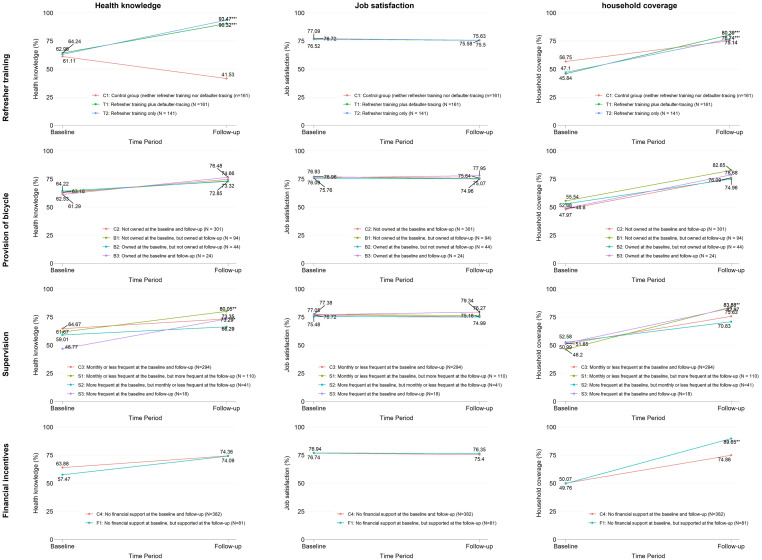
Changes of community healthy workers’ performance indicators between baseline and follow-up surveys by the intervention groups.

Figure 1 presents the trends of three CHWs’ performance indicators by the intervention groups. Also, the supplementary file reported the detail information (i.e., values and standard errors) for each point in Figure 1. Unadjusted DID estimate for each intervention were calculated (Table 2). Only training (T1) and training plus defaulter tracing (T2) increased CHWs’ health knowledge against no training group (C1) by 45.7% and 50.1%, respectively, and similarly increased their household coverage by 16.2% and 11.3%, respectively. Compared with CHWs having been supervised by CHEWs monthly or less frequently as of both baseline and follow-up surveys (C3), those having been supervised monthly or less frequently as of the baseline survey but more frequently as of the follow-up survey (S1) increased their health knowledge (9.7%) and household coverage (13.0%). Similarly, compared with CHWs having been supervised by CHEWs monthly or less frequently, those having more frequently supervised as of the follow-up survey than the baseline survey (S3) increased their health knowledge by 17.8% and visited a greater number of households in their catchment communities by 6.7%. CHWs having received financial incentives (F1) increased their health knowledge (6.2%) and visited a greater number of households (15.1%) than those not having (C4).

The results of fixed effect regressions for respective performance variables were shown in Table 3. Of all types of single and combined interventions, three interventions significantly increased in CHWs’ health knowledge, that is: (i) refresher training plus defaulter tracing [Coef.: 38.80, 95% CI: 32.71–44.90, *P* < 0.001]; (ii) refresher training only [Coef.: 48.43, 95% CI: 42.09–54.76, *P* < 0.001]; and (iii) combination of refresher training plus defaulter tracing and frequent supervision [Coef.: 17.02, 95% CI: 7.90–26.15, *P* < 0.001]. Financial support was the only intervention that significantly increased job satisfaction among CHWs [Coef.: 4.97, 95% CI: 0.20–9.75, *P* = 0.041]. There was no single intervention that significantly increased household coverage. Yet, the combinations of the interventions significantly increased household coverage. That is, refresher training plus defaulter tracing and provision of a bicycle [Coef.: 14.47, 95% CI: 5.65–23.29, *P* = 0.001]; refresher training plus defaulter tracing and financial support [Coef.: 25.99, 95% CI: 15.00–36.98, *P* < 0.001]; refresher training and provision of a bicycle [Coef.: 14.47, 95% CI: 5.23–23.70, *P* = 0.002]; refresher training and frequent supervision [Coef.: 21.64, 95% CI: 10.86–32.42, *P* = 0.001]; refresher training and financial support [Coef.: 19.11, 95% CI: 8.15–30.07, *P* < 0.001].

**Table 3.** Results of the fixed effect model to assess the effectiveness of the interventions and the combinations in community health workers (CHWs’) performances in Nyanza Province, Kenya

	(1)	(2)	(3)
	Health knowledgeCoef. (95% CI)	Job satisfactionCoef. (95% CI)	Household coverageCoef. (95% CI)
Time	-19.39 (-23.64–-15.14)^***^	-2.362 (-3.985–-0.740)^**^	19.36 (14.39–24.32)^***^
**Interventions**			
Training plus DT^†^	38.80 (32.71–44.90)^***^	1.739 (-0.622–4.099)	5.666 (-1.666–13.00)
Training only	48.43 (42.09–54.76)^***^	1.808 (-1.053–4.668)	-4.284 (-12.71–4.147)
Provision of Bicycle	1.504 (-3.195–6.202)	-0.284 (-2.582–2.014)	-5.435 (-11.23–0.361)
Frequent supervision	-3.628 (-9.508–2.253)	-0.455 (-2.541–1.631)	4.050 (-2.099–10.20)
Financial supports	-1.298 (-9.916–7.320)	4.974 (0.203–9.746)^*^	-2.537 (-10.48–5.407)
**Interactions**			
Training plus DT^†^ × Bicycle	4.031 (-3.950–12.01)	-2.026 (-7.863–3.811)	14.47 (5.652–23.29)^**^
Training plus DT^†^ × Supervision	17.02 (7.899–26.15)^***^	1.610 (-2.218–5.438)	5.882 (-4.446–16.21)
Training plus DT^†^ × Financial support	6.651 (-5.499–18.80)	-6.362 (-13.54–0.820)	25.99 (15.00–36.98)^***^
Training × Bicycle	-1.454 (-9.269–6.362)	-0.464 (-4.311–3.384)	14.47 (5.231–23.70)^**^
Training × Supervision	3.913 (-4.944–12.77)	-0.724 (-4.241–2.793)	21.64 (10.86–32.42)^**^
Training × Financial support	11.40 (-0.403–23.20)	-5.073 (-10.83–0.685)	19.11 (8.154–30.07)^***^

* *P* < 0.05.

† Defaulter tracing activity.

** *P* < 0.01.

*** *P* < 0.001.

All models were controlled by sex, age, marital status, education status, wealth index, sanitation practice and working year as CHWs.

It was found that the year of conducting survey was negatively influenced in the score of health knowledge [Coef.: -19.39, 95% CI: -23.64–-15.14, *P* < 0.001] and job satisfaction [Coef.: -2.36, 95% CI: -3.99–-0.74, *P* = 0.004], while household coverage significantly increased over time [Coef.: 19.36, 95% CI: 14.39–24.32, *P* < 0.001].

## Discussion

This study attempts to assess the effectiveness of four interventions and their combinations in CHWs’ performances represented by three key proxy variables (i.e., health knowledge, job satisfaction, and household coverage).

Health knowledge is one of the most important capacities to deliver appropriate and quality health messages to community members. This study found that refresher training, regardless of addition of defaulter tracing, was an effective intervention for increasing CHWs’ health knowledge. This is consistent with the positive association between training and CHWs’ knowledge reported by a number of earlier studies (Harvey *et al*., 2008; Amare, 2009; Kane *et al*., 2010; Lopes *et al.*, 2014). Repeated and continued refresher training on a monthly basis during the period from January to July of 2012 were provided to CHWs. Generally, impacts and effectiveness of refresher training were gradually reduced over time, unless follow-up refresher training or supportive supervision was undertaken (Lopes *et al.*, 2014). Therefore, it is recommended that follow-up refresher training be regularized to keep CHWs knowledgeable on health issues. The use of training materials composed exclusively of basic and visualized contents is the key to ensuring the effectiveness of training program for CHWs. When revising or redesigning the training materials to further increase the effectiveness of refresher training, CHWs’ sociodemographic and socioeconomic characteristics such as education and literacy levels should be sensitively considered. This is because the sociodemographic and socioeconomic characteristics are one of critical determinants of CHWs’ understanding on health knowledge and health-service performances (Ande *et al*., 2004; Kawakatsu *et al.*, 2015). The combination of monthly refresher training plus defaulter tracing and >1 supportive supervisions of CHWs per month is likely to have created a greater synergetic effect in increasing and sustaining CHWs’ health knowledge than other combinations. Onsite supportive supervision by CHEWs would supplement what is missed or inadequate in classroom-type refresher training, for example, by reminding CHWs of the contents of refresher training and by providing CHWs with opportunities to have individual-based question and answer sessions. Thus, supportive supervisions by CHEWs twice a month or more might have served as an effective supplement to the basic core intervention (i.e., refresher training plus defaulter tracing).

An earlier study in Ghana reported that health workers’ job satisfaction and motivation were significantly associated with willingness to stay at current duty stations (Bonenberger *et al*., 2014). Two other studies in African countries reported that insufficient remuneration was one of the major reasons for CHWs’ attrition (Gray and Ciroma, 1988; Kironde and Klaasen, 2002). Our study found that provision of financial incentives was the only intervention that improved CHWs’ job satisfaction. The interventions without financial incentives were likely not only to reduce job satisfaction but also to often result in CHWs’ more frequent turnovers and replacements. On the other hand, several earlier studies reported that financial incentives damaged CHWs’ intrinsic motivation (Glenton *et al.*, 2010) and that inadequate financial incentives rather discourage CHWs from performing well (Greenspan *et al*., 2013). Thus, both pros and cons of provision of financial incentives should be thoroughly considered in advance, when designing and planning for performance-based financial incentives for CHWs. Note that performance-based financing always involves a certain risk to significant challenges in sustainability, unless its stable and regular budget line is secured.

This study suggests that only specific combinations of refresher training and supplementary interventions were effective in increasing household coverage by CHWs’ visits. An earlier study interestingly reported that CHWs need to be provided not either but both of financial and nonfinancial incentives, to enable them to effectively work (Abdel-All *et al.*, 2019). This study demonstrated that a combination of refresher training and provision of bicycle would encourage CHWs having greater health knowledge to visit a greater proportion of households. This is most likely because the burden of house-to-house walking was alleviated by using a bicycle as transport means. Other nonfinancial incentives such as social recognitions (e.g., certifying and awarding) and provision of commodities to be used for CHWs’ activities (e.g., bags and T-shirts) were also effective in improving CHWs’ performances (Amare, 2009; Abdel-All *et al.*, 2019). It is worth noting synergetic effects between monthly refresher training and financial incentives in increasing household coverage. Frequent supervision along with monthly refresher training produced a positive impact on household coverage. This finding is in line with the results of several earlier studies (Kane *et al.*, 2010; Laínez *et al.*, 2012; Greenspan *et al.*, 2013). Yet, that combination might have produced no impact on their performances, if frequent supervision had not been implemented to respond to and address CHWs’ poor performance. That could be one of the reasons that a significant effect was not identified in the combination of training, defaulter tracing, and supervision.

In sum, the results of our study indicate that CHWs’ performances represented by three proxies (i.e., health knowledge, job satisfaction, and household coverage) are expected to increase, when having implemented both monthly refresher training and financial incentive provision. Cascaded and community-arranged training design, as described in the method section, could help not only ensure sustainability of refresher training through its cost reduction but also enhance communities’ ownership of health activity through gradually building the trust between CHWs and local populations. As recommended by an earlier review work (Kaschko, 2014), regulation of CHWs’ wage at either national or subnational level is indispensable for sustaining the CHW program by pre-empting possible overpayment of remunerations. The wage gap between CHWs would create unnecessary sense of unfairness among them and subsequently likely lead to compromised performances and higher attrition rate. This study found that financial incentive alone would not improve all the aspects of CHWs’ performances. Combining two types of interventions (e.g., monthly refresher training and financial incentives) is recommended, to accelerate CHWs performances and eventually improve populations’ health status.

All the three key proxy performance indicators fluctuated over time regardless of the interventions and independent variables, as shown in Table 3. Regarding the reduction of the health knowledge over time, the main reason would be that the open-ended questions of health knowledge for follow-up survey included a greater number of questions than that for baseline survey. It resulted in the lower average score in the follow-up survey among the CHWs without the refresher training. When no intervention was made (i.e., control group), CHWs’ job satisfaction declined over time among CHWs of control groups for whom no intervention was implemented. Conversely, household coverage increased over time among them. The Kenyan MoH announced the increase in the number of households per CHW. This policy change has been requiring district health departments to reduce the number of CHWs per CU. It is recommended that better performing CHWs be strategically selected, when implementing the policy. This would help to minimize the possible risks to reduction in household coverage.

### Limitations

This study attempted to estimate the effectiveness of four different interventions and their combinations, using the longitudinal data representative of CHWs in the study sites. However, there are several limitations in this study. First, this study assumed that unobserved time-varying variables were independent of the observed time-varying variables (i.e., the four interventions). Any different types of interventions other than the four this study addresses have been neither observed in nor reported from the study sites. Therefore, our assumption should be reasonable. Second, this study employed only the three types of key proxy performance outcomes (i.e., health knowledge, job satisfaction, and household coverage), since they are commonly recognized performance of CHWs in the earlier studies. Yet, probably, several other types of performance outcomes should be employed in the future studies, such as those related to community-based treatments. Exclusion of the data collected from 228 CHWs having only partially responded to the questionnaires from analysis might have biased the data analysis results to a certain extent.

## Conclusions

The combinations of regular refresher training and other intervention(s) are the recommended as the effective interventions in improving and further sustaining CHWs’ performances such as health knowledge, job satisfaction, and household coverage. Refresher training is likely to have helped increase, update, and retain CHWs’ health knowledge, while financial support is independently likely to have helped increase their job satisfaction. The combinations are the effective interventions to increase household coverage.

## Supplementary material

For supplementary material accompanying this paper visit http://doi.org/10.1017/S1463423622000135. click here to view supplementary material
