# Supplementary material for: Effectiveness of four interventions in improving community health workers’ performance in western Kenya: a quasi-experimental difference-in-differences study using a longitudinal data
Source: Prim Health Care Res Dev. 2022 Mar 25;23:e20. doi: 10.1017/S1463423622000135 (PMC8991856; doi:10.1017/S1463423622000135)
Supplement: Supplementary file 1 [file phcsup.zip › S1463423622000135sup001.docx]

| **Supplement file. Results of univariate analysis between the outcome and control variables** | | | | | | | | | | | | |
| --- | --- | --- | --- | --- | --- | --- | --- | --- | --- | --- | --- | --- |
|  | **Health Knowledge** | | | | **Job Satisfaction** | | | | **Household Coverage** | | | |
|  |  | 95% CI | |  |  | 95% CI | |  |  | 95% CI | |  |
| **Variable** | Coef. | low | high | p-value | Coef. | low | high | p-value | Coef. | low | high | p-value |
| **Gender** |  |  |  |  |  |  |  |  |  |  |  |  |
| Female | Ref. |  |  |  | Ref. |  |  |  | Ref. |  |  |  |
| Male | 1.150 | -3.118 | 5.418 | 0.598 | -0.804 | -1.965 | 0.357 | 0.175 | 1.719 | -2.005 | 5.443 | 0.366 |
| **Age** |  |  |  |  |  |  |  |  |  |  |  |  |
| Younger than 30 years of age | Ref. |  |  |  | Ref. |  |  |  | Ref. |  |  |  |
| 30 – 39 years of age | -3.507 | -9.482 | 2.468 | 0.251 | -0.598 | -2.242 | 1.046 | 0.476 | -2.873 | -8.132 | 2.386 | 0.285 |
| 40 years of age or older | -7.921 | -13.807 | -2.034 | 0.009 | -0.556 | -2.176 | 1.064 | 0.501 | -4.172 | -9.353 | 1.01 | 0.115 |
| **Marital Status** |  |  |  |  |  |  |  |  |  |  |  |  |
| Married | Ref. |  |  |  | Ref. |  |  |  | Ref. |  |  |  |
| Others | -3.135 | -7.477 | 1.207 | 0.158 | 0.407 | -0.778 | 1.592 | 0.501 | 1.000 | -2.797 | 4.797 | 0.606 |
| **Educational Status** |  |  |  |  |  |  |  |  |  |  |  |  |
| No education or not completed primary education | Ref. |  |  |  | Ref. |  |  |  | Ref. |  |  |  |
| Primary education | 7.735 | 1.669 | 13.8 | 0.013 | 1.498 | -0.165 | 3.162 | 0.078 | 5.268 | -0.071 | 10.607 | 0.054 |
| Secondary education or higher | 11.000 | 4.276 | 17.724 | 0.001 | 1.886 | 0.042 | 3.731 | 0.046 | 4.824 | -1.094 | 10.743 | 0.111 |
| **Wealth Index** |  |  |  |  |  |  |  |  |  |  |  |  |
| Poorest | Ref. |  |  |  | Ref. |  |  |  | Ref. |  |  |  |
| Poor | 2.454 | -3.938 | 8.847 | 0.452 | -0.152 | -1.898 | 1.594 | 0.865 | 0.286 | -5.294 | 5.866 | 0.920 |
| Middle | -0.836 | -6.286 | 4.614 | 0.764 | -0.481 | -1.970 | 1.008 | 0.527 | 2.650 | -2.107 | 7.407 | 0.275 |
| Rich | 0.970 | -4.188 | 6.129 | 0.713 | -0.111 | -1.520 | 1.298 | 0.878 | 0.857 | -3.645 | 5.360 | 0.709 |
| Richest | -2.674 | -8.167 | 2.818 | 0.340 | 0.137 | -1.363 | 1.638 | 0.858 | 3.646 | -1.148 | 8.440 | 0.137 |
| **Availability of Sanitation Facilities** |  |  |  |  |  |  |  |  |  |  |  |  |
| No facility available | Ref. |  |  |  | Ref. |  |  |  | Ref. |  |  |  |
| One or two facilities available | 2.333 | -3.883 | 8.549 | 0.462 | 1.334 | -0.340 | 3.007 | 0.119 | 0.263 | -5.181 | 5.707 | 0.925 |
| All three facilities available | 4.934 | -0.896 | 10.764 | 0.098 | 2.720 | 1.151 | 4.289 | 0.001 | 0.558 | -4.547 | 5.663 | 0.831 |
| **Working year as CHWs** |  |  |  |  |  |  |  |  |  |  |  |  |
| Less than four years | Ref. |  |  |  | Ref. |  |  |  | Ref. |  |  |  |
| Four years or longer | 0.238 | -3.275 | 3.751 | 0.895 | 0.252 | -0.705 | 1.209 | 0.606 | 0.657 | -2.409 | 3.723 | 0.675 |
