# Supplementary material for: Effectiveness of four interventions in improving community health workers’ performance in western Kenya: a quasi-experimental difference-in-differences study using a longitudinal data
Source: Prim Health Care Res Dev. 2022 Mar 25;23:e20. doi: 10.1017/S1463423622000135 (PMC8991856; doi:10.1017/S1463423622000135)
Supplement: Supplementary file 1 [file phcsup.zip › S1463423622000135sup002.docx]

| **Supplement table 2. Associations matrix between the interventions and control variables** | | | | | |
| --- | --- | --- | --- | --- | --- |
| **Demographic and socio-economic characteristics** | **Training plus DT^1^** | **Training only** | **Provision of Bicycle** | **Frequent supervision** | **Financial supports** |
|  | p-value | p-value | p-value | p-value | p-value |
| Gender | 0.210 | 0.021* | <0.001* | 0.133 | 0.236 |
| Age | 0.615 | 0.417 | 0.225 | 0.338 | 0.413 |
| Marital Status | 1.000 | 0.348 | 0.506 | 0.829 | 0.574 |
| Educational Status | 0.097 | 0.157 | 0.435 | 0.135 | 0.926 |
| Wealth Index | 0.105 | 0.138 | <0.001* | 0.136 | 0.858 |
| Availability of Sanitation Facilities | 0.867 | 0.115 | 0.233 | 0.368 | 0.381 |
| Working year as CHWs | 0.699 | 0.139 | 0.784 | 0.112 | 0.124 |
| Associations between the two variables were assessed based on Chi-Square test.  *** p<0.001, ** p<0.01, * p<0.05  ^1^ Defaulter tracing activity | | | | | |
